# Electric field tuning of a nickel zinc ferrite resonator by non-linear magnetoelectric effects

**DOI:** 10.1038/s41598-023-45530-4

**Published:** 2023-10-26

**Authors:** Maksym Popov, Alexander Machi, Jerad Inman, Rao Bidthanapally, Sujoy Saha, Hongwei Qu, Menka Jain, Michael R. Page, Gopalan Srinivasan

**Affiliations:** 1 Institute of High Technologies, Taras Shevchenko National University of Kyiv, Kyiv, 01601 Ukraine; 2 Department of Physics, Oakland University, Rochester, MI 48309 USA; 3 Electrical and Computer Engineering Department, Oakland University, Rochester, MI 48309 USA; 4 Department of Physics, University of Connecticut, Storrs, CT 06269 USA; 5 Materials and Manufacturing Directorate, Air Force Research Laboratory, Wright-Patterson Air Force Base, Dayton, OH 45433 USA

**Keywords:** Materials science, Physics

## Abstract

The nature of nonlinear magnetoelectric (NLME) effect has been investigated at room-temperature in a single-crystal Zn substituted nickel ferrite. Tuning of the frequency of magnetostatic surface wave (MSSW) modes under an applied pulsed DC electric field/current has been utilized to probe the effect. The frequencies of the modes at 8–20 GHz were found to decrease by ~ 400 MHz for an applied DC power *P* of ~ 100 mW and the frequency shift was the same for all of the MSSW modes and linearly proportional to *P*. A model is proposed for the effect and the NLME phenomenon was interpreted in terms of a reduction in the saturation magnetization due to the DC current. The decrease of magnetization with applied electric power, estimated from data on mode frequency versus *P*, was − 2.50 G/mW. The frequency tuning efficiency of the MSSW modes due to NLME effects in the ferrite resonator was found to be 4.1 MHz/mW which is an order of magnitude higher than the shift reported for M-type strontium and barium hexaferrite resonators investigated earlier. The spinel ferrite resonator discussed here has the potential for miniature, electric field tunable, planar microwave devices for the 8–20 GHz frequency range.

## Introduction

Microwave devices such as resonators and band-stop and band-pass filters are important components in high frequency communication devices and radars^1–3^. Such devices, in general, are based on low-loss semiconductors, ferroelectrics, or ferrites^1–6^. Tuning the operating frequencies of ferroelectric devices could be accomplished with an electric field *E* and the devices could easily be miniaturized. The frequency of ferrite-based devices, however, could only be tuned with a source of variable magnetic field *H*^4–8^. Thus, ferrite microwave devices cannot be miniaturized or integrated with semiconductor devices due to the need for a solenoid or similar sources to generate a variable *H*. A significant development in microwave device technologies is the use of a ferrite-ferroelectric composite to facilitate *E* tuning of the ferrite devices^9–12^. The *E*-tuning is possible due to magneto-electric (ME) effect is such composites^13, 14^. The piezoelectric strain under an *E*-field in the composite when transferred to the ferrite manifests as an induced magnetic field and leads to tuning of the ferrite device^12–15^. A variety of dual *H*- and *E*-tunable ferrite-ferroelectric devices including resonators, filters, and inductors were demonstrated in the past years^16–20^.

An alternate *E*-tuning mechanism of ferrite devices aided by non-linear ME effect (NLME) was reported recently^21, 22^. The NLME effect, first reported in M-type strontium hexagonal ferrite, was observed as tuning of ferromagnetic resonance (FMR) in the ferrite under a DC or pulsed *E*-field. The *E/I*-induced changes in the magnetization and anisotropy field due to NLME, determined from FMR data, were found to vary with the input DC power (or *E*^*2*^). Studies on *c*-plane single crystals of (Sr, Al)- and Ba-hexaferrites also revealed a similar NLME effects for currents either in the *c*-plane or along the *c*-axis^23, 24^. *E*- tunable resonators and filters of M- and Y-type hexagonal ferrites operating at 12–50 GHz were demonstrated^25–27^.

This report is on the first observation of a similar NLME effect in a spinel ferrite with cubic symmetry. We present here experimental evidence for the effect in a microwave resonator with a single crystal platelet of nickel zinc ferrite. The device operates in the X- to K-bands, thus complements similar resonators and filters that make use of hexaferrites for higher frequencies. Besides, nickel ferrite easily adopts to dopants such as Zn which makes possible tuning of its magnetic parameters for ferrite devices operating at a specific frequency range^28^. The experiments were carried out with the ferrite in a stripline device structure and a static magnetic field *H*_*0*_was applied to excite magnetostatic surface wave (MSSW) modes in the 8–20 GHz frequency range^29, 30^. An in-plane DC current applied parallel to *H*_*0*_ resulted in a down shift in the mode frequencies of equal magnitude for all of the modes. The decrease in the mode frequency ∆*f* was in excess of 400 MHz for a DC power *P* ~ 400 mW and ∆*f* was found to vary linearly with *P*. Data on mode frequency *f* versus *P* was used to estimate the changes in the magnetic parameters for the ferrite. Since the frequency shift due to NLME effects is similar to the influence of sample heating, experiments carried out to determine the Joule heating effect indicated a frequency shift that was an order of magnitude smaller than the shift due to NLME effects. Details on our studies are provided in the sections that follow.

## Experiment

**Figure 1:**
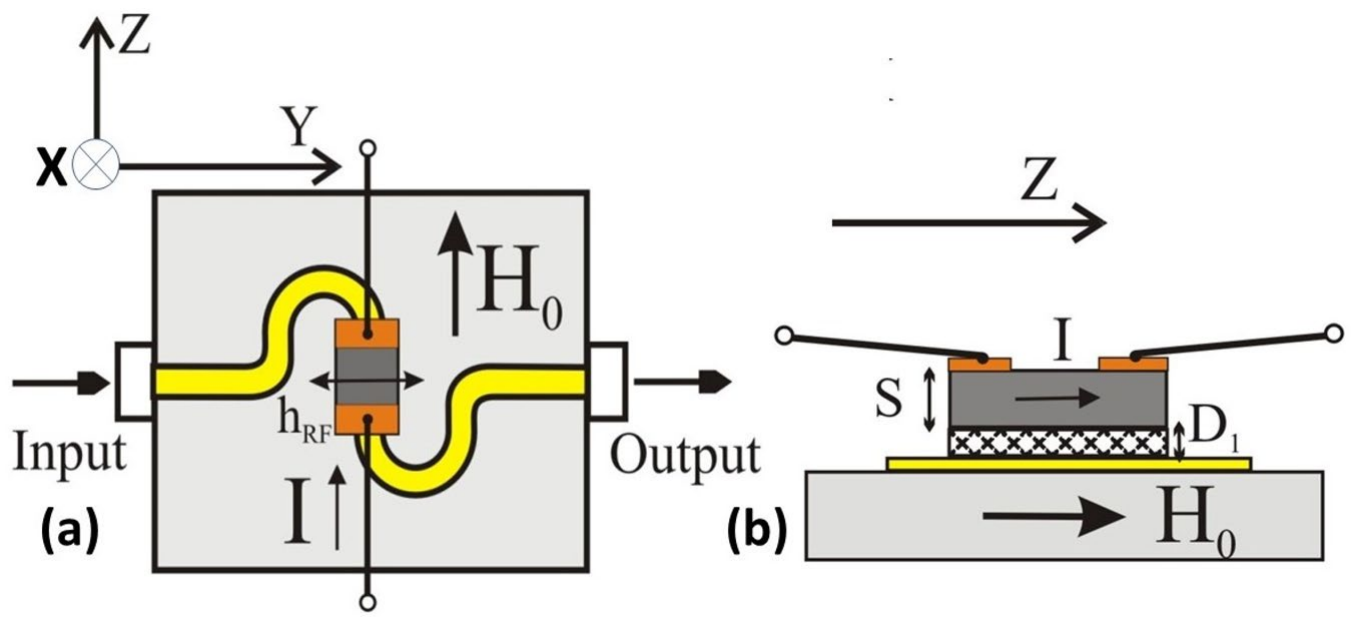
(**a**) Schematic diagram showing the experimental setup with the nickel zinc ferrite sample, Ni_0.7_Zn_0.3_Fe_2_O_4_ (NZFO), with Pt-electrodes placed on top of a microstrip line. (**b**) Cross section view of the NZFO samples. S is the sample thickness and D_1_ is the thickness of an adhesive epoxy layer. The static field *H*_*0*_ and the DC current *I* are applied along the Z-direction.

The material under investigation was a single-crystal platelet of zinc-substituted nickel ferrite with the composition Ni_0.7_Zn_0.3_Fe_2_O_4_ (NZFO) grown by floating zone technique (and provided by Professor A. Balbashov, Moscow Power Engineering Institute, Russia). It was cut into rectangular slabs in such way that the crystallographic < 111 > direction coincided with the normal to the sample plane. It was then polished down to a thickness of *S* = 335 μm and to the lateral dimensions *a* × *b* = 2.5 × 1 mm^2^. Finally, conducting electrodes (3 μm of *Pt* on 45 nm *Ti* buffer layer) as shown in Fig. 1 were deposited on opposite sides of samples by magnetron sputtering. Those electrodes provided a path for current flow through the NZFO in the (111) plane along the longer side. Figure 1 shows the experimental setup with the NZFO sample placed on top of a microstrip transmission line. The current *I* is parallel to the static field *H*, along the *Z*-direction. The sample is separated from the stripline by an adhesive dielectric layer of thickness *D*_1_*.*

**Figure 2:**
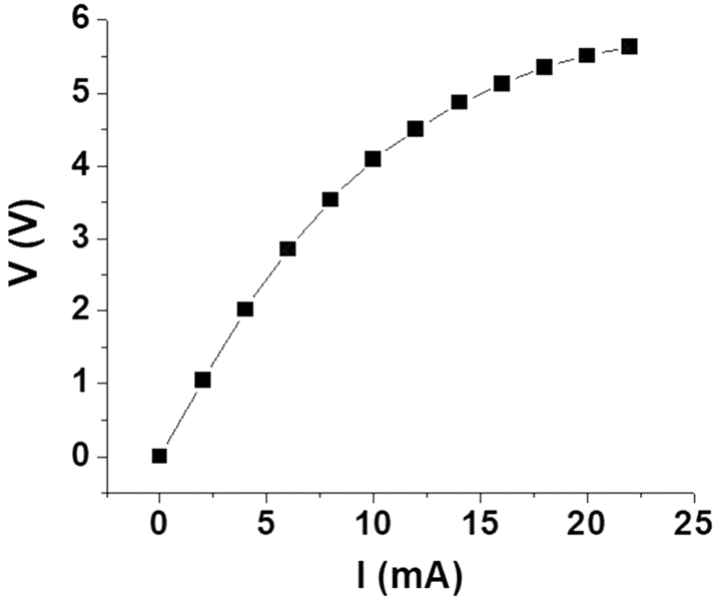
DC voltage *V* versus current *I* data for the NZFO sample.

Since the DC electric field/current is a key parameter in the measurements, the voltage *V* versus current *I* characteristics were measured for the sample and are shown in Fig. 2. The current *I* varies linearly with *V* for *V* < 2 V, corresponding to a resistance *R* ~ 510 Ω., and a nonlinear variation in *I* is evident for higher *V* values.

## Results

**Figure 3:**
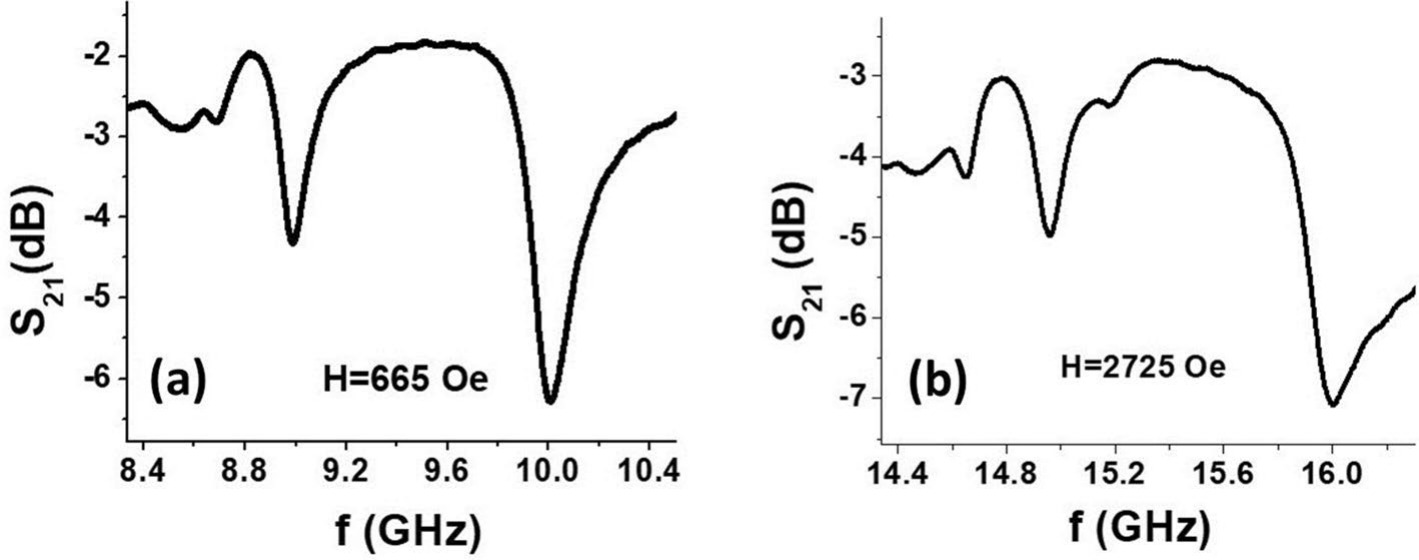
Transmission loss *S*_*21*_ as a function of frequency *f* for NZFO sample for an in-plane static magnetic field (**a**) *H* = 665 Oe and (**b**) 2725 Oe. The profiles show two dominant magnetostatic surface wave (MSSW) modes.

**Figure 4:**
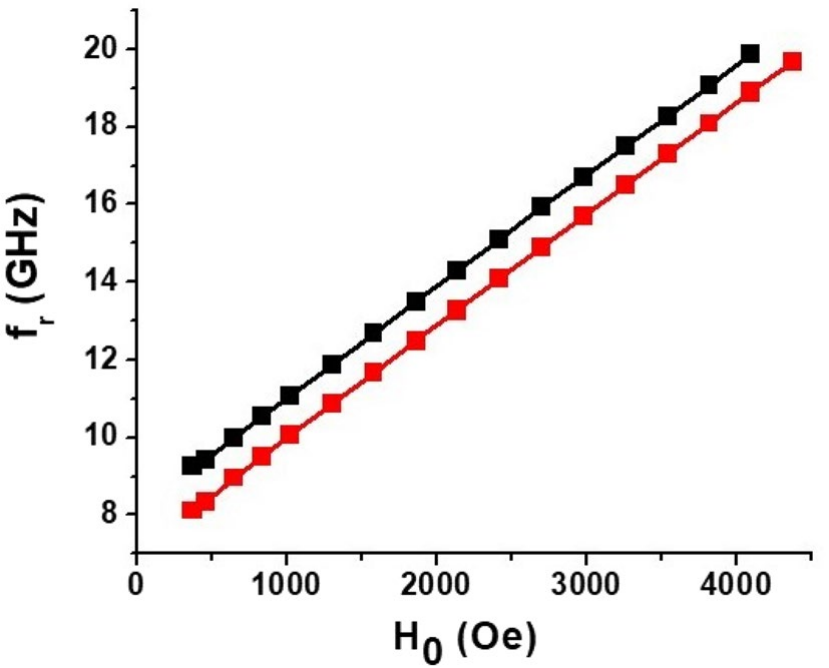
MSSW mode frequency *f*_*r*_ as a function of the applied magnetic field *H*_0_ for the two dominant modes as in Fig. 3.

The NZFO sample was placed on top of a S-shaped microstrip line etched on a 0.02 inch (508 μm) thick RT/Duroid 5880 substrate. A thin adhesive tape of thickness of thickness *D*_*1*_ = 20 μm was used to fix the sample in place. A bias magnetic field *H*_0_ was applied along the longer side of the sample for the excitation of MSSW modes in the ferrite. The stripline structure with the NZFO sample was connected to the ports of a vector network analyzer (Agilent PNA E8361A) and excited with microwave power (Figure S-1). The scattering matrix parameter*S*_21_for the resonator was measured as a function of the frequency *f* of the input microwave power. Representative *S*_*21*_ versus *f* profiles for *H* = 665 Oe and 2725 Oe are shown in Fig. 3. Depending on *H*_*0*_ values, several MSSW modes were observed in the frequency range 8–16 GHz. The two dominant modes are shown in Fig. 3. Similar measurements were done for a series of *H*_*0*_ values ranging from 400 to 4400 Oe (corresponding to *f*_*r *_≈ 8–20 GHz) (Figure S-2). Data on the variation of *f*_*r*_ with *H* obtained from such profiles are shown in Fig. 4 (and also in Figure S-3).

**Figure 5:**
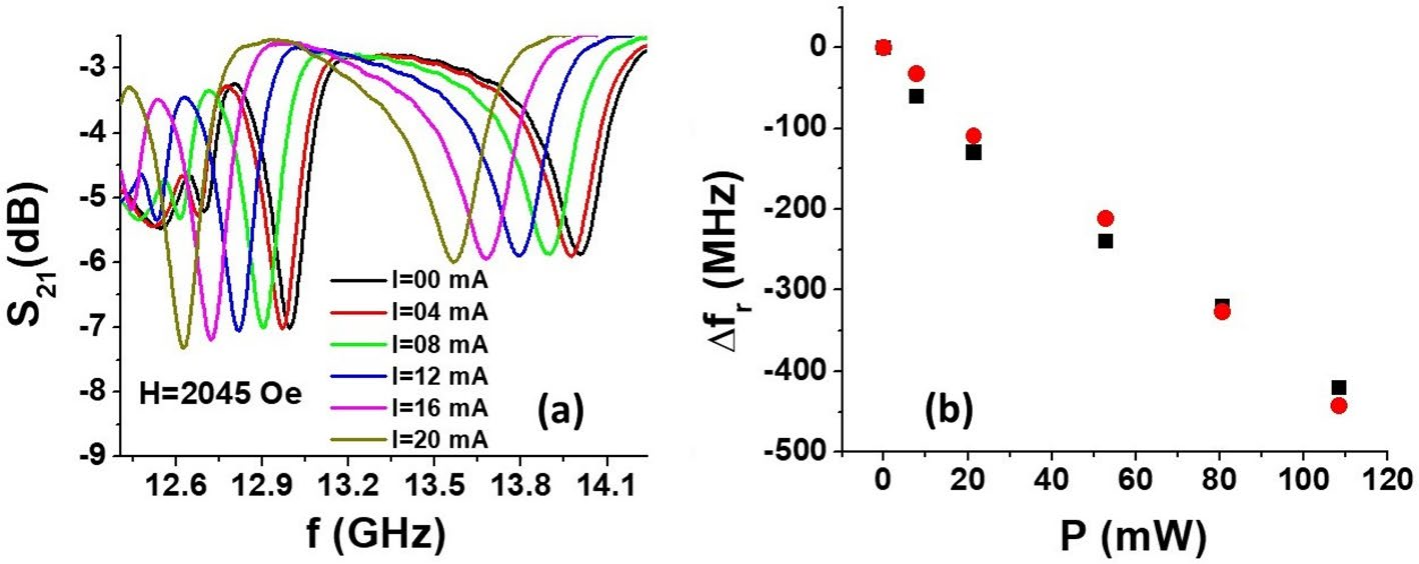
(**a**) Profiles of S_21_ versus f showing the current tuning of the dominant resonance modes in the NZFO platelet for *H* = 2045 Oe for *I* = 0–20 mA. (**b**) Variation in the *I*-induced shift ∆*f*_*r*_ in the lower (squares) and higher (circles) frequency modes with the input DC power *P*.

Following the measurements of *f*_*r*_ versus *H*_0_, studies were carried out on the tuning of the resonator mode frequency with a DC *E*/*I* for constant *H*_0_ values. A DC voltage single pulse, 1–5 s in duration, applied to the electrodes (shown in Fig. 1), established a current parallel to *H*_0_. Successive pulses were applied after a time interval of 1 min to minimize potential heating of the sample. Figure 5 shows a series of *S*_*21*_ versus *f* profiles for the two dominant MSSW modes for *H*_0_ value of 2045 Oe and *I* = 0–20 mA. The *E*/*I*-induced shift in the mode frequency ∆*f*_*r*_ is also shown for the two modes as a function of the input power *P.* It is clear from the results that ∆*f*_*r*_ for the two modes are approximately the same for a specific *P*-value. Both modes show a decrease in the frequency shift with increasing input power and their dependence on *P* is linear. Similar linear variation in ∆*f*_*r*_ was measured for a series of bias magnetic fields as shown in Fig.S-4–S-7 in the supplement. From now on we, therefore, focus only on results obtained for the higher frequency MSSW mode.

**Figure 6:**
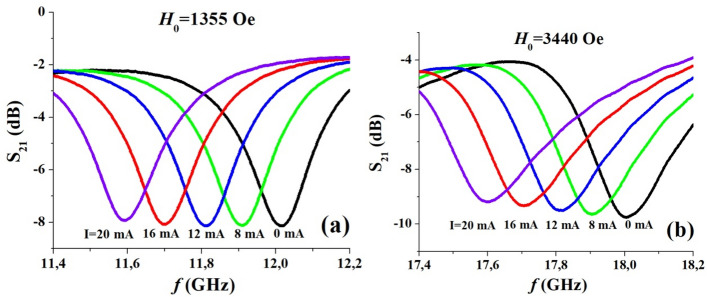
Representative data for current tuning of the high frequency MSSW modes in (111) NZFO resonator for the current flowing parallel to in-plane magnetic field *H*_*0*_.

**Figure 7:**
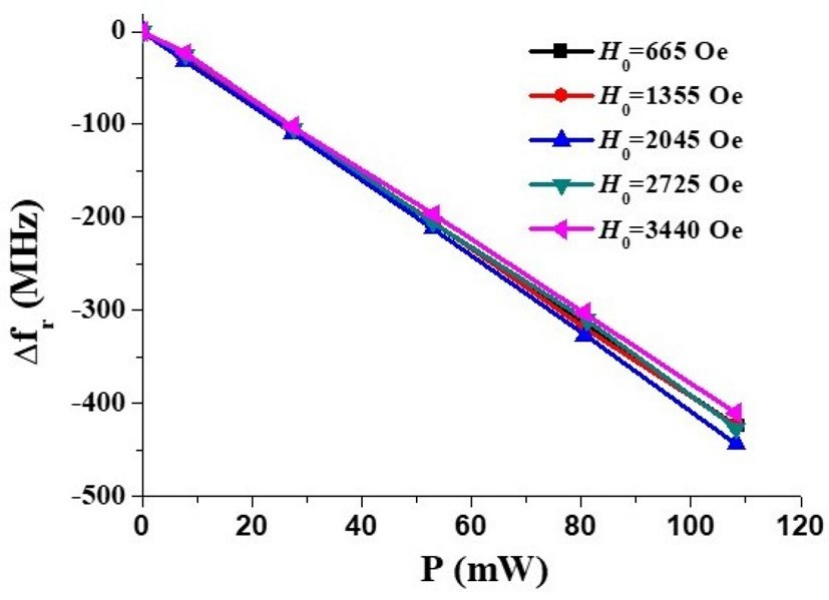
Shift in the MSSW mode frequency versus applied electric power measured for a series of magnetic bias field values.

Results on the *I*-tuning of the high frequency mode are shown in Fig. 6 for *H*_0_ = 1355 Oe and 3440 Oe and the variation in the mode frequency shift ∆*f*_*r*_ with *P* is shown in Fig. 7 for a series of *H*_0_ values. It is evident that the measured decrease in ∆*f*_*r*_ with *P* is independent for *H*_0_ value. It is also obvious from the data in Fig. 7 that the *E*-tuning of the mode frequency is due to nonlinear ME effects and ∆*f*_*r*_ is proportional to *E*^*2*^ (and not just *E*). The data also allows one to extract the efficiency of tuning, the slope Δ*f*_*r*_/∆*P*, for meaningful comparison with other materials or electrically tunable ferrite devices. The tuning efficiency is Δ*f*_*r*_/∆*P* ~ 4.1 MHz/mW for the NZFO resonator. Comparison of the tuning efficiency with similar ferrite devices are provided in later sections.

## Discussion

Linear ME effects mediated by mechanical deformation have been studied extensively in composites of ferrites and ferroelectrics^12–14^. Due to symmetry considerations such linear ME effects, however, are forbidden in ferrites with collinear magnetic ordering. A theory for the NLME effects observed in both hexagonal^21–27^ and spinel ferrites is lacking at present. One may attribute the NLME to weakening of the magnetic interactions due to the exclusion of electrons involved in carrying a current in the ferrite under a DC electric field. Even though stoichiometric spinel ferrites are expected to be insulators, divalent Fe are generally present in them due to impurities. Hopping type conduction in the ferrites, therefore, leads to a semiconductor like behavior. Under an applied DC voltage, electrons in Fe sites involved in carrying a current are likely to be excluded from contributing to super-exchange interactions. Weakening of the magnetic interactions will lead to a decrease in the overall magnetization of the ferrite. That exclusion will be in effect for during the time electrons hop from one iron site to another. One may, therefore, attribute the decrease in the magnetization that manifests as a decrease in the frequency of resonance modes to weakening of magnetic exchange interactions and consequent decrease in the magnetization.

We first discuss a model for the MSSW modes and their *E*-tuning in a uniformly magnetized ferrite platelet enclosed between conducting metal layers, with ferrite to metal spacings equal to D_1_ and D_2_^30^. The explict dispersion equation for this case is given by Eq. (6) in Ref.^30^ which is:

1 $$\begin{aligned} & (1 + \kappa )(1 + \kappa + \eta^{2} ) - \left( {\nu - \sqrt {1 + \eta^{2} } \tanh \left( {\frac{{D_{1} }}{S}\varsigma \sqrt {1 + \eta^{2} } } \right)} \right)\left( {\nu + \sqrt {1 + \eta^{2} } \tanh \left( {\frac{{D_{2} }}{S}\varsigma \sqrt {1 + \eta^{2} } } \right)} \right) \\ & \quad + (1 + \kappa )\sqrt {1 + \eta^{2} } \sqrt {1 + \frac{{\eta^{2} }}{1 + \kappa }} \left( {\tanh \left( {\frac{{D_{1} }}{S}\varsigma \sqrt {1 + \eta^{2} } } \right) + \tanh \left( {\frac{{D_{2} }}{S}\varsigma \sqrt {1 + \eta^{2} } } \right)} \right)\coth \left( {\varsigma \sqrt {1 + \frac{{\eta^{2} }}{1 + \kappa }} } \right) = 0 \\ \end{aligned}$$

where $\kappa = \Omega_{H} /(\Omega_{H}^{2} - \Omega^{2} )$, $\nu = \Omega /(\Omega_{H}^{2} - \Omega^{2} )$, $\Omega_{H} = H_{0} /\left( {4\pi M_{0} } \right)$, $\Omega = \omega /\left( {\gamma 4\pi M_{0} } \right)$, $\xi = k_{z} S$, $\zeta = k_{y} S$, and $\eta = \xi /\zeta = k_{z} /k_{y}$. Here 4*πM*_0_ is the saturation magnetization of the ferrite, γ = 2.8 MHz/Oe^28^ is the gyromagnetic ratio. The coordinate system used here is shown on the Fig. 1.

In our experimental set-up the bottom metal layer is the central stripe of the S-shaped microstrip line (Fig. 1) of width same as the resonator width *b*. The distance between the bottom metal layer and the ferrite is 20 μm which is the thickness of the adhesive layer. The top metal layer is formed by the *Pt* electrodes that partially cover the sample surface. In essence, the ferrite sample consists of two regions with different MSSW dispersion characteristics $\omega (k)$. The first one is the middle area with only the bottom metal screen. The dispersion equation for this region can be obtained from Eq. (1) by substituting the screen spacing D_1_ = 20 μm and D_2_ = ∞, respectively:

2 $$\begin{aligned} & (1 + \kappa )(1 + \kappa + \eta^{2} ) - (\nu - {\text{sgn}} (\varsigma )\sqrt {1 + \eta^{2} } )\left( {\nu + \sqrt {1 + \eta^{2} } \tanh \left( {\frac{{D_{2} }}{S}\varsigma \sqrt {1 + \eta^{2} } } \right)} \right) \\ & \quad + (1 + \kappa )\sqrt {1 + \eta^{2} } \sqrt {1 + \frac{{\eta^{2} }}{1 + \kappa }} \left( {{\text{sgn}} (\varsigma ) + \tanh \left( {\frac{{D_{2} }}{S}\varsigma \sqrt {1 + \eta^{2} } } \right)} \right)\coth \left( {\varsigma \sqrt {1 + \frac{{\eta^{2} }}{1 + \kappa }} } \right) = 0 \\ \end{aligned}$$

The second region is the ferrite under the *Pt* electrodes. This case is described by conditions D_1 _= 20 μm and D_2 _= 0, which leads to the following dispersion equation^29, 30^

3 $$\begin{aligned} & (1 + \kappa )(1 + \kappa + \eta^{2} ) - \nu \left( {\nu + \sqrt {1 + \eta^{2} } \tanh \left( {\frac{{D_{2} }}{S}\varsigma \sqrt {1 + \eta^{2} } } \right)} \right) \\ & \quad + (1 + \kappa )\sqrt {1 + \eta^{2} } \sqrt {1 + \frac{{\eta^{2} }}{1 + \kappa }} \tanh \left( {\frac{{D_{2} }}{S}\varsigma \sqrt {1 + \eta^{2} } } \right)\coth \left( {\varsigma \sqrt {1 + \frac{{\eta^{2} }}{1 + \kappa }} } \right) = 0 \\ \end{aligned}$$

**Figure 8:**
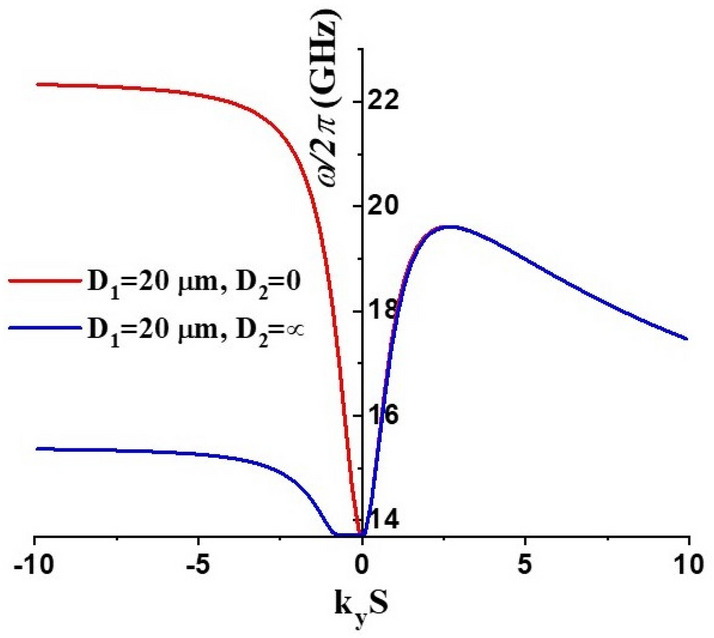
Nonreciprocal dispersion characteristic of MSSW in an isotropic ferrite layer calculated using Eqs. (1) and (2) and for *H*_0_ = 3000 Oe, *4πM*_0_ = 5000 G and *ξ* = 1. Note, that for the positive values of wavevector (k_y_ > 0) the dispersion curves for D_2_ = 0 and D_2_ = ∞ are nearly identical (frequency difference is less than 150 MHz for the given parameters).

[all the notations are the same as in Eq. (1)]. The above equations are valid for an isotropic ferromagnet and thus can be applied in practice only in the situation when the anisotropy field is negligible in comparison to both the static magnetization and *H*_*0*_ which is true for the NZFO used in this study^28^. The presence of the metal screen leads to a pronounced nonreciprocity of the MSSW propagation, namely, the waves with the opposite directions of the wave-vector will have different frequencies: $\omega (k) \ne \omega ( - k)$. That fact strongly influences the resonator modes since they are usually formed by the interference of the counter-propagating waves. An example for the nonreciprocal dispersion characteristics of the MSSW in both regions of the resonator is shown in the Fig. 8.

In order to determine the resonance frequencies of the ferrite one needs to define the in-plane wave-vector set by the geometric dimensions of the resonator. The sign of longitudinal wave-vector *k*_*z*_ (or $\xi$) (with respect to the *H*_0_ direction) does not affect the dispersion Eqs. (2) or (3) and therefore it can be calculated as

4a $$k_{z} = m\frac{\pi }{d},m = 0,1,2...$$

Here *d* is the length of the metal electrode (*d ≈ *0.7 mm in our case) or the distance of metal-free region between the electrodes. On the other hand, the transversal wave-vector $k_{y}$ due to the nonreciprocity of MSSW should be evaluated from the condition^31^

4b $$(k_{y}^{ + } + |k_{y}^{ - } |)b = 2\pi n,\,\,n = 0,1,2...$$

where $k^{ \pm }$ are the positive and negative solutions of the Eq. (2) or (3) and *b* is the width of the sample. The modes represented by Eqs. (4a) and (4b) will therefore be labeled as (*m, n*) modes. The observed FMR frequency variation is given by

5 $$\Delta f_{r} = \frac{{\partial f_{r} }}{{\partial (4\pi M_{0} )}}\Delta (4\pi M_{0} )$$

Assuming that Δ(4*πM*_0_) is *linearly* proportional to the applied power^21–24^, the expected shift *Δf*_*r*_ also should be proportional to *P* which agrees with the results in Fig. 7. One may then use the above theory to extract the NLME induced variation in the saturation magnetization. For the nickel-zinc ferrite of the given composition 4π*M*_0 _≈ 5000 G whereas the cubic anisotropy field *H*_*a*_ is less than 100 Oe^28^. Therefore, the condition 4π*M*_0_ >  > *H*_*a*_ is fulfilled and use of Eqs. (2), (3) is justified.

The higher frequency mode shown in Fig. 3 was identified as (1, 1) mode of the metalized ferrite region. We used this mode to estimate the NLME induced changes in magnetization from ∆*f*_*r*_ versus *P* data in Fig. 7. The following procedure was used for the estimate. For specific *f*_*r*_ and 4π*M*_0_ ≈ 5000 G, γ = 2.8 MHz/Oe, and the dimensions of the resonator (*d, b, S*) the wave-vector values $k^{ \pm }$ were calculated from Eq. (2). Then the values of $k^{ \pm }$ were substituted into Eq. (4b) and checked whether this equation was satisfied. If not, then the procedure was repeated for series of values of 4π*M*_0_ until the wave vector values satisfied both Eq. (3) and Eq. (4b). This procedure was repeated for *f*_*r*_ corresponding to different values of *P* to determine the variation of 4π*M*_0_ with *P.* The results on 4π*M*_0_ versus *P* for a series of *H*_*0*_ values are shown in Fig. 8. We took only the data for the largest values of external bias field since in this case the influence of static demagnetization field (that was not taken into account in Eq. (1)–(3) ) is expected to be minimal. One notices a linear decrease in 4π*M*_0_ at an average rate of − (2.50 ± 0.12) G/mW for all of the *H*_*0*_ values. The deviation in 4π*M*_0_ at *P* = 0 from the expected value of 5000 G may be attributed to the contribution from the cubic anisotropy field *H*_a_ ~ 100 Oe^29^. Additionally, one may expect a variation in the cubic anisotropy field on *E*^32^ due to NLME effects which was not considered in the model discussed here.

**Figure 9:**
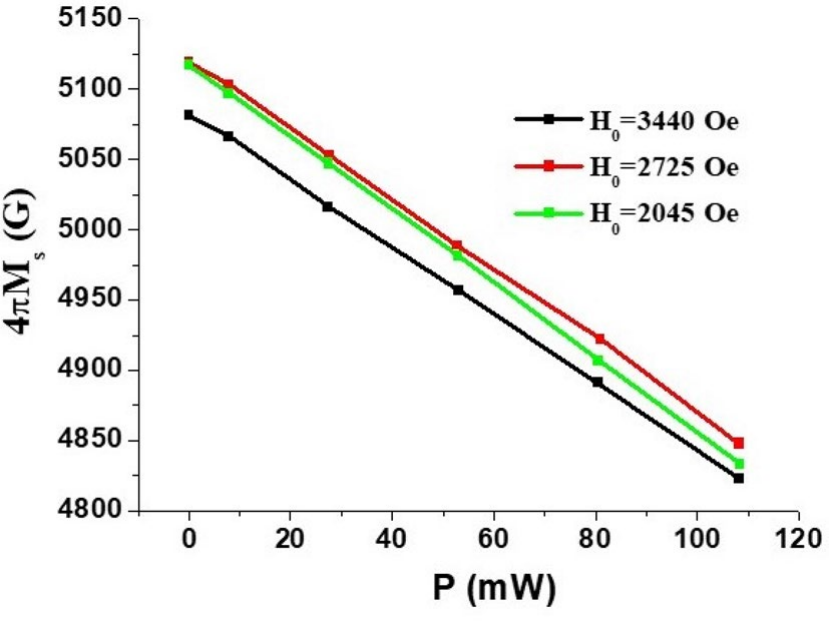
Calculated dependence of the nickel-zinc ferrite saturation magnetization $4\pi M_{0}$ on the applied dc electric power *P*.

**Figure 10:**
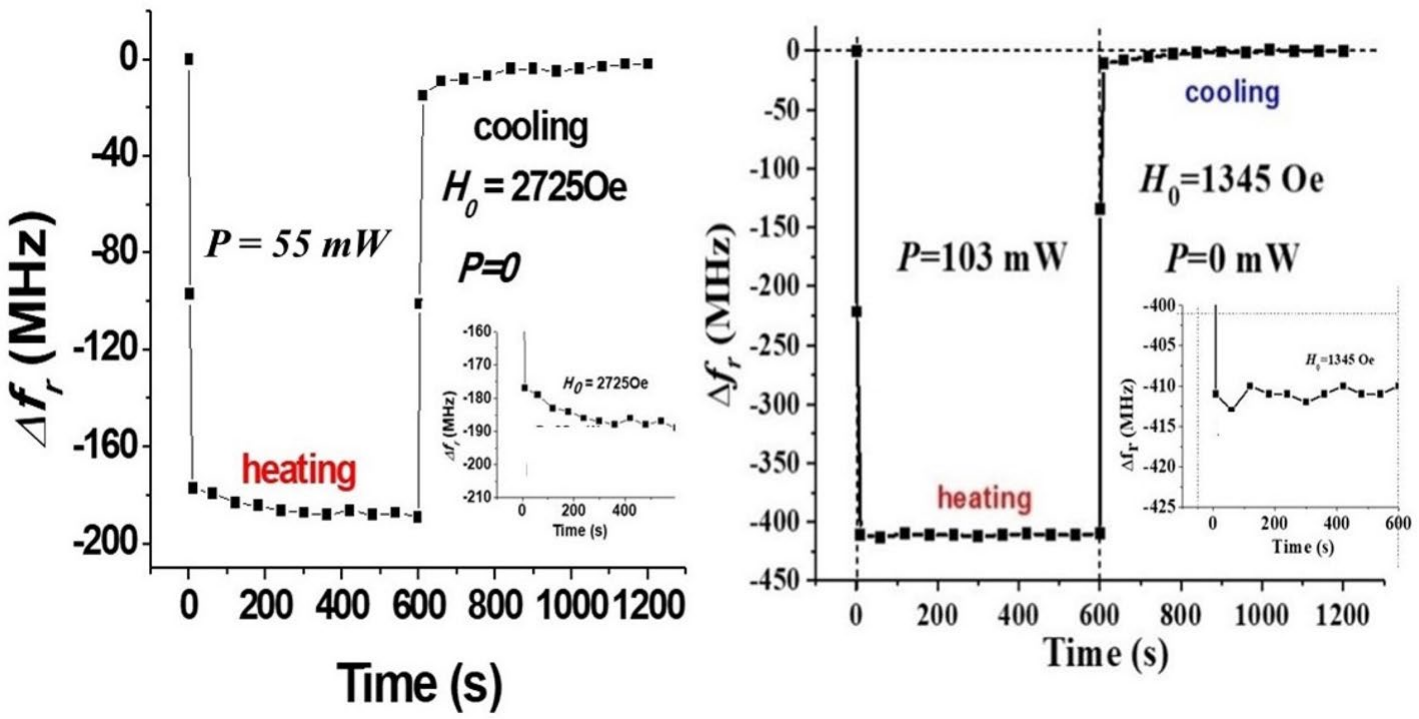
Data on Magnetic mode frequency shift under a prolonged application of a continuous DC current for the input power (**a**) P = 55 mW and (**b**) 103 mW.

In the course of the measurements the DC voltage/current was applied as short pulses to the nickel ferrite sample in order to eliminate the effect of Joule heating of the sample and its contribution to any changes in *f*_*r*_. The pulses were of duration 1–5 s. It is important to investigate the effects of sample heating compared to the influence of NLME effects. Additional experiments were done to separate these two effects on the decrease in the mode frequency under a DC current. Although the data provided in Figs. 3, 4, 5, 6, 7 and 9 (in the revised version) are for pulsed currents of duration 1–5 s, we examined the effects of sample heating under a *continuous DC current*. The current, corresponding to input power P = 55 mW and 103 mW, was first turned on for a duration of 10 min and the resonance frequency f_r_ was recorded at regular time intervals. The current was then turned off after 10 min, and data on f_r_ versus t were recorded for the next 10 min. The results on ∆f_r_ versus t are shown in Fig. 10. For both DC powers, ∆f_r_ shows an abrupt decrease by 180 MHz for P = 55 mW and by 410 MHz for P = 103 MHz as soon as the current is on and then shows a further decrease by 5–15 MHz over the 10 min period that the current was present. As soon as the current is turned off, f_r_ shows an abrupt increase and ∆f_r_ slowly decays to zero during the next 10 min of cooling. It is therefore evident from these experiments that Joule heating results only in 4–8% of the overall shift due to NLME effects.

The ferrite platelet in our experiments had a large surface area-to-volume ratio that is favorable in terms of effective heat exchange. The top surface was exposed to air and the bottom surface with a thin adhesive layer is on top of the thermally conducting copper microstrip line. If the sample were thermally isolated and under a prolonged application of a continuous DC current, the temperature (and thus mode frequency) should continuously change with time. But data in Fig. 10 shows that this is not the case. Next, such thermally isolated sample should remain at high temperature after the current was switched off and thus the resonance frequency should vary slowly after the power being switched off. But, f_r_ shows an immediate jump, and regains its the initial value as the sample cools down. It is safe to conclude from the data in Fig. 10 that any contribution due to sample is small compared to NLME effects.

The nature of strain mediated nonlinear ME effects in ferrite-ferroelectric composites was studied in detail and well understood. But there have been very few reports so far on NLME in spinel or hexagonal ferrites^21–27^. We compare the strength of NLME in the spinel ferrite with similar reports for hexagonal ferrites. As mentioned earlier, the MSSW mode frequency tuning efficiency from Fig. 7 for the NZFO resonator Δ*f*_*r*_/∆*P *≈ − 4.1 MHz/mW (or alternatively, Δ*P*/Δ*f*_*r*_ = − 0.25mW/MHz). In the case of M-type barium hexaferrite Δ*f*_*r*_/Δ*P* ≈ + 0.27 MHz/mW^25^. The efficiency was + 0.36 MHz/mW for *Al*-substituted M-type strontium hexaferrite^11^ and − 14.2 MHz/mW for Y-type hexaferrite^26^. Hence, the tuning efficiency for NZFO is higher than reported values for M-type hexaferrites, but not as high as for Y-type hexaferrite.

## Conclusions

Evidence for electric field/current-induced NLME effects at room-temperature in single crystal nickel zinc ferrite resonator were obtained from studies on frequency tuning of MSSW modes at 8–20 GHz. It was found that the application of an in-plane DC current resulted in a down-shift in the MSSW mode frequencies that was linearly dependent on the DC electric power. This proportionality to *P* implies a nonlinear, quadratic in electric field *E*, nature of the observed ME effect and is similar to our earlier observations in M- and Y-type hexaferrites. The shift in mode frequencies due to NLME was independent of the mode frequency and was attributed to a reduction in the saturation magnetization of the ferrite. Using a rigorous theory for the MSSW modes in the ferrite slab with metal shielding, we were able to determine the variation of saturation magnetization with applied DC power. It was found that in all cases the magnetization decreased linearly with *P* at a rate of − 2.50 G/mW. The NLME related frequency tuning efficiency was 4.1 MHz/mW which is higher than the reported values for M-type hexaferrites with uniaxial anisotropy, but smaller than in Y-type hexaferrite with easy plane anisotropy. For the traditional *H*-tunable YIG filter the frequency tuning is linear with respect to the current in the solenoid coil and the tuning efficiency was 4–4.5 MHz/mW in the 10–18 GHz range^33^. The results in this study indicate the utility of the NLME phenomenon in the spinel ferrite for planar, energy efficient electrically tunable microwave signal-processing devices that can be miniaturized and integrated with semiconductor devices.

### Supplementary Information

Supplementary Figures.

## Data Availability

The datasets used and/or analyzed during the current study will be available from the corresponding author upon reasonable request.
