# Supplementary material for: Electric field tuning of a nickel zinc ferrite resonator by non-linear magnetoelectric effects
Source: Sci Rep. 2023 Oct 26;13:18346. doi: 10.1038/s41598-023-45530-4 (PMC10603105; doi:10.1038/s41598-023-45530-4)
Supplement: Supplementary file 1 — Supplementary Figures. [file 41598_2023_45530_MOESM1_ESM.pdf]

Supplementary to :

Electric Field Tuning of a Nickel Zinc Ferrite Resonator by Non-Linear Magnetoelectric Effects

Maksym Popov,[1,2] Alexander Machi,[2] Jerad Inman,[3] Rao Bidthanapally,[2] Sujoy Saha,[2] Hongwei Qu,[3] Menka Jain,[4] Michael R. Page,[5] and Gopalan Srinivasan[2*]

[1] Institute of High Technologies, Taras Shevchenko National University of Kyiv, Kyiv 01601, Ukraine

[2] Department of Physics, Oakland University, Rochester, Michigan 48309, USA

[3] Electrical and Computer Engineering Department, Oakland University, Rochester, Michigan 48309, USA

[4]Department of Physics, University of Connecticut, Storrs, Connecticut 06269, USA

[5] Materials and Manufacturing Directorate, Air Force Research Laboratory, Wright-Patterson Air Force Base, Dayton, Ohio 45433, USA

* Corresponding author email: srinivas@oakland.edu

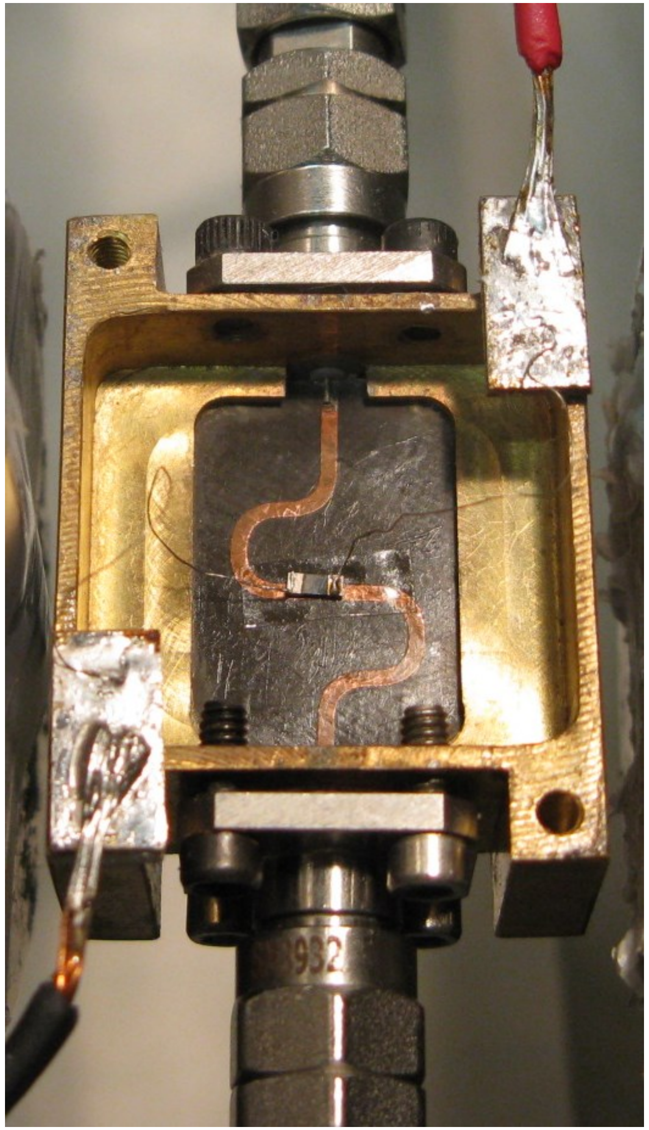

Fig.S-1: The photo of experimental setup, showing S-shaped microstrip transmission line with (111) NZFO resonator atop on it. The attached wires are used to supply DC electric current to the ferrite sample.

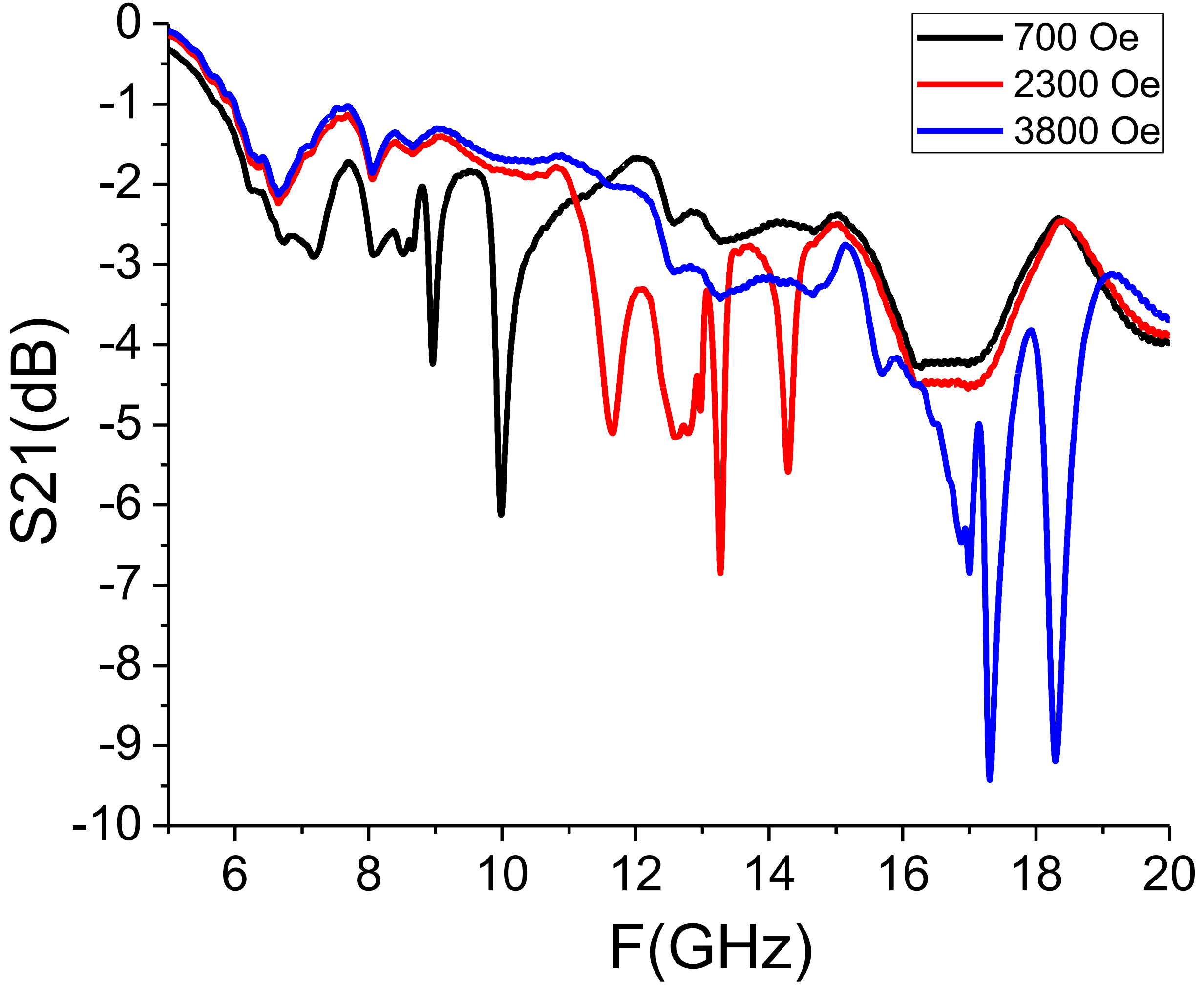

Fig.S-2: The transmission characteristics of NZFO resonator measured in the 5-20 GHz frequency range for three different values of the in-plane bias magnetic field.

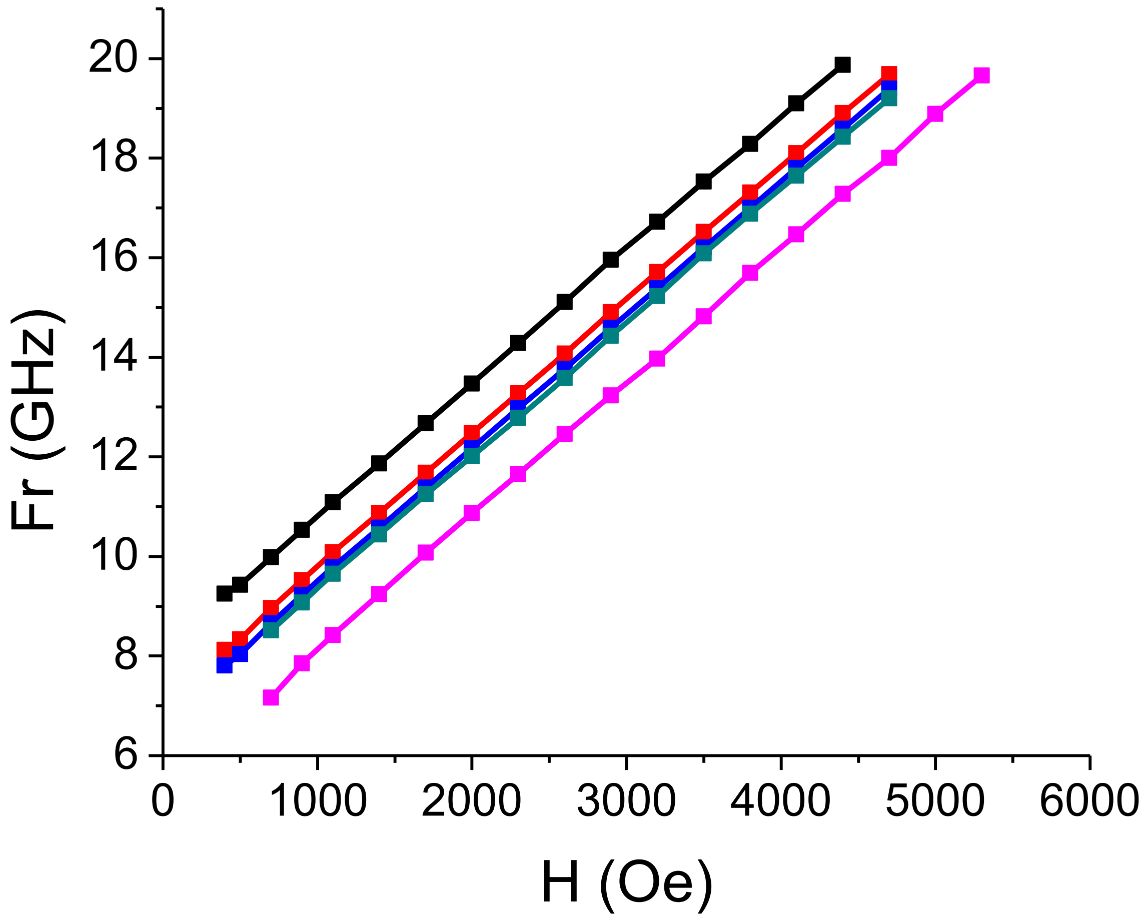

Fig.S-3: The frequency vs. magnetic field dependence for the observed magnetostatic surface wave (MSSW) modes of the NZFO rectangular ferrite resonator.

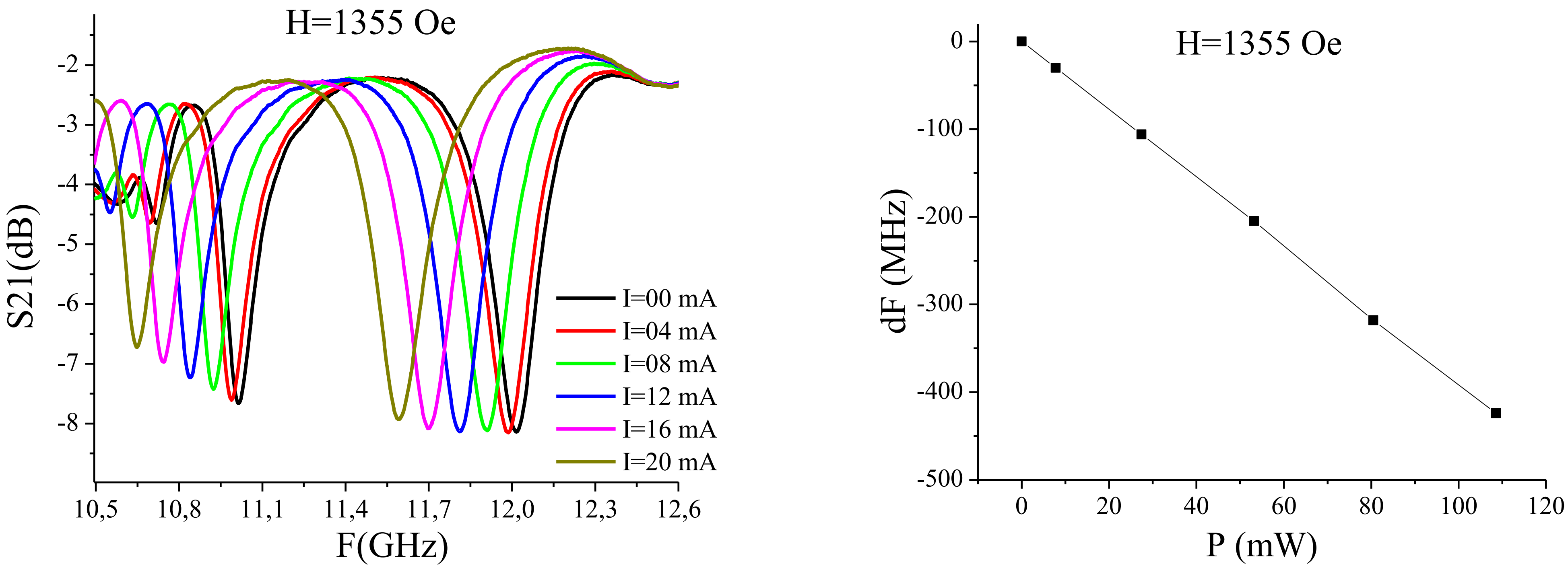

Fig.S-4: : (left) Representative profiles of S21 vs f showing current tuning of the MSSW modes in the (111) NZFO resonator for the current parallel to in-plane magnetic field H_0 = 1355 Oe. (**right**) Data on shift in the mode frequency as a function of applied DC power for the highest frequency mode.

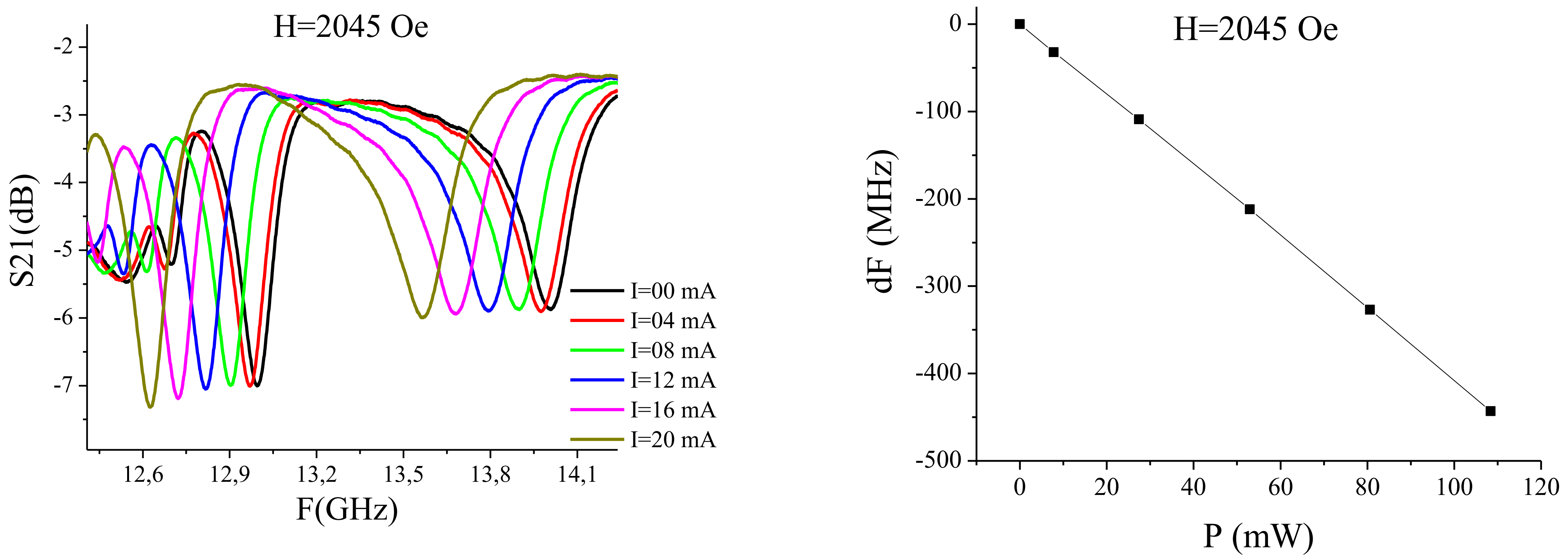

Fig.S-5: : (left) Representative profiles of S21 vs f showing current tuning of the MSSW modes in the (111) NZFO resonator for the current parallel to in-plane magnetic field H_0 = 2045 Oe. (**right**) Data on shift in the mode frequency as a function of applied DC power for the highest frequency mode.

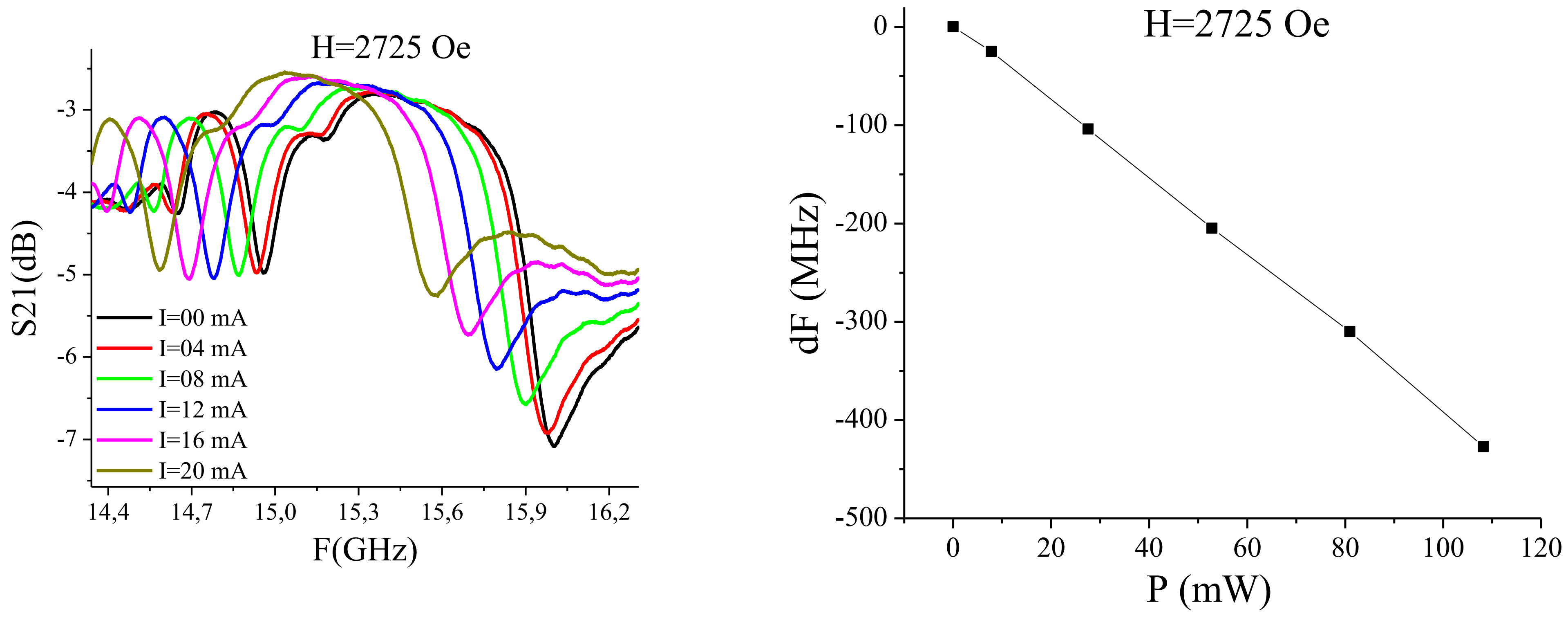

Fig.S-6: : (left) Representative profiles of S21 vs f showing current tuning of the MSSW modes in the (111) NZFO resonator for the current parallel to in-plane magnetic field H_0 = 2725 Oe. (**right**) Data on shift in the mode frequency as a function of applied DC power for the highest frequency mode for H_0 = 2725 Oe.

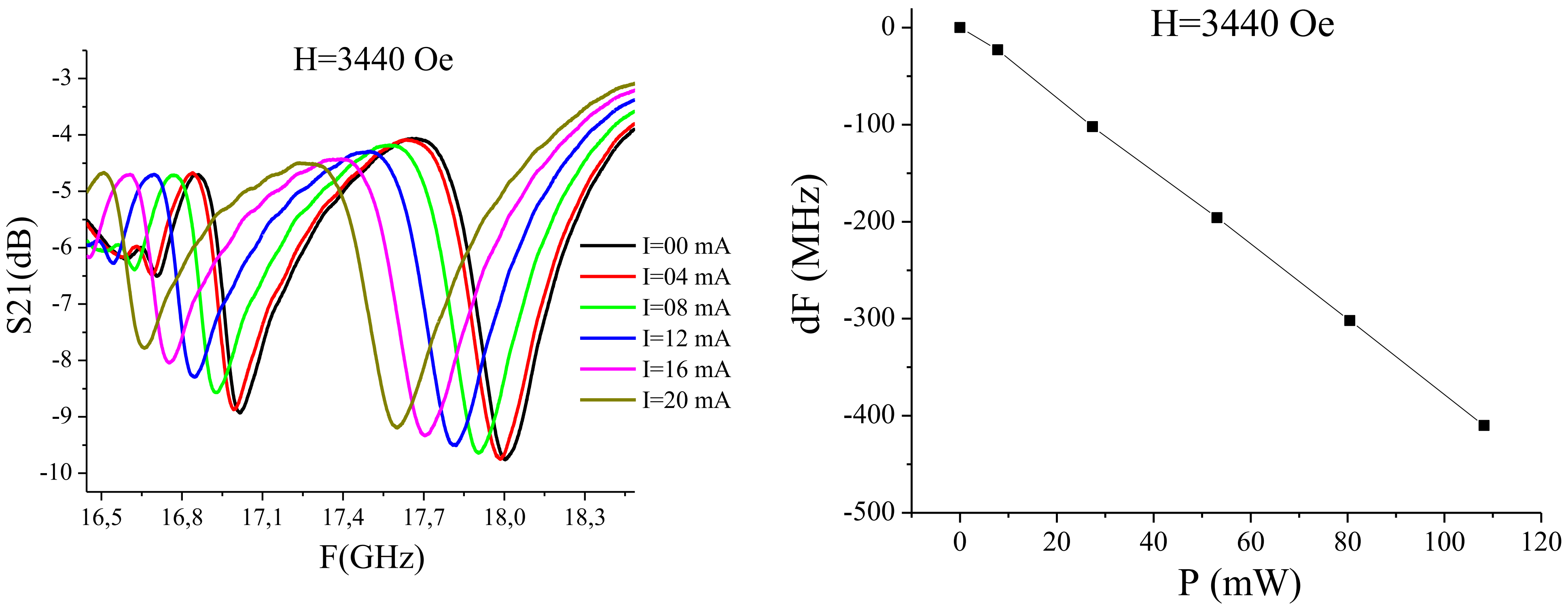

Fig.S-7: : (left) Representative profiles of S21 vs f showing current tuning of the MSSW modes in the (111) NZFO resonator for the current parallel to in-plane magnetic field H_0 = 3440 Oe. (**right**) Data on shift in the mode frequency as a function of applied DC power for the highest frequency mode. H_0 = 3440 Oe.
